# Promoting the inclusion of Afghan women and men in research: reflections from research and community partners involved in implementing a ‘proof of concept’ project

**DOI:** 10.1186/s12939-015-0145-3

**Published:** 2015-01-31

**Authors:** Elisha Riggs, Jane Yelland, Josef Szwarc, Sue Casey, Donna Chesters, Philippa Duell-Piening, Sayed Wahidi, Fatema Fouladi, Stephanie Brown

**Affiliations:** Healthy Mothers Healthy Families Research Group, Murdoch Childrens Research Institute, Flemington Road, 3052 Parkville, VIC Australia; General Practice and Primary Health Care Academic Centre, University of Melbourne, 3052 Parkville, VIC Australia; Victorian Foundation for Survivors of Torture, 4 Gardiner Street, 3056 Brunswick, VIC Australia; School of Population and Global Health, University of Melbourne, 3052 Parkville, VIC Australia

**Keywords:** Health inequalities, Community engagement, Partnerships, Refugee health

## Abstract

**Introduction:**

With mounting evidence that poor maternal and child health outcomes are related to the social determinants of health, researchers need to engage with vulnerable and isolated communities to gather the evidence that is essential to determine appropriate solutions. Conventional research methods may not ensure the degree and quality of participation that is necessary for meaningful study findings. Participatory methods provide reciprocal opportunities for often excluded communities to both take part in, and guide the conduct of research.

**Method/design:**

The *Having a baby in a new country* research project was undertaken to provide evidence about how women and men of refugee background experience health services at the time of having a baby. This two year, multifaceted proof of concept study comprised: 1) an organisational partnership to oversee the project; 2) a community engagement framework including: female and male Afghan community researchers, community and sector stakeholder advisory groups and community consultation and engagement.

**Discussion:**

Inclusive research strategies that address power imbalances in research, and diversity of and within communities, are necessary to obtain the evidence required to address health inequalities in vulnerable populations. Such an approach involves mindfully adapting research processes to ensure that studies have regard for the advice of community members about the issues that affect them. Researchers have much to gain by committing time and resources to engaging communities in reciprocal ways in research processes.

## Introduction

In high-income countries such as Australia, population groups most likely to have the greatest need for high quality health care are often the least well served by health services [1]. Racial and ethnic disparities in health outcomes have been extensively documented, with much of this literature focusing on the accessibility of services, and whether patients receive appropriate tests, procedures and medications [2,3]. Increasingly, questions are being asked about the quality of social interactions between health professionals and minority populations [2,4].

People with refugee backgrounds comprise a growing proportion of the Australian population. Under the Humanitarian program, Australia accepts 13,750 refugees per year, the majority are children, and men and women of child bearing age. The experiences and living conditions that refugee women have endured in their countries of origin and on their journey to their new country of settlement – unattended births, traumatic and unsafe abortions, use of unsterilized equipment, poor sanitation, female genital mutilation/cutting and high rates of fetal death in utero and infant mortality - contribute to the risk of obstetric complications and may cause women to be fearful and anxious about receiving maternity care [5]. The circumstances leading to people fleeing their own country - experiences of persecution, or having a well-founded fear of persecution – mean that many live with ongoing trauma symptoms, and the accumulative stressors of settlement, and loss or separation from family members.

Whilst a few international [6,7] and Australian studies have investigated refugee women’s experiences of maternity care [8,9] there is limited evidence of how Afghan refugee families including men, access and navigate the Australian maternity and early childhood health care systems and the responsiveness of these health services to refugee families. Local evidence suggests that refugee women are less likely to attend the recommended number of antenatal check-ups, and more likely to attend hospital emergency departments for obstetric complications [10] and face barriers to access and remain engaged with maternal and child health services [11].

The *Having a baby in a new country: The experiences of Afghan families and stakeholders* project was initiated in response to a lack of information about the experiences of women and men from refugee backgrounds using maternity and early childhood services. The results of this study have been published [12]. This project was conceived as a ‘proof of concept’ study involving a community engagement framework with participatory research strategies. The project was designed to generate new knowledge about: the experiences of refugee women and men having a baby in a new country; engaging and consulting with refugee communities; and methodological approaches ‘that work’ in hearing the voices of refugee families.

The aims of this paper are: (i) to report the steps that we took in developing the *Having a baby in a new country* project, and (ii) to describe some of the challenges and ethical dilemmas associated with implementing community and health service engagement strategies; and (iii) to reflect on lessons learned from the study.

## Forming the partnership and designing the proof of concept study

The project developed out of discussions between researchers at the Healthy Mothers Healthy Families research group at the Murdoch Childrens Research Institute (MCRI) and staff at the Victorian Foundation for Survivors of Torture (Foundation House) commencing in 2010. Foundation House is an agency that provides services and support for people of refugee backgrounds who have fled persecution, torture and war-related trauma to find safety in Australia. Our early discussions focused on what was known about maternal and child health outcomes for refugee families, how families experience and navigate Australian maternity services, and the limitations of available research to inform system reform.

A partnership was formed between the MCRI and Foundation House to explore ways that we could work together to address these gaps in the evidence to inform strengthening of services for families of refugee background. In the beginning we spent time considering how the two organisations would work together to carry out the work of the project. This included discussion of values and principles for undertaking this kind of project; clarifying our respective roles with regard to the design, conduct and interpretation of the research; and careful consideration of the ethical aspects of conducting research with people of refugee background.

The involvement of the Afghan community in south eastern Melbourne was selected by the partnership group. It was thought that the salient issues for individual communities are likely to not only be related to the refugee experience but also to cultural and community context. In addition it was thought that if the methods were deemed appropriate, the study could be repeated with other refugee populations.

It was at this stage of the project that several ethical issues critical to the context of engaging refugee background families in maternity and early childhood research were identified and given careful consideration. In particular, we discussed: (i) the possibility that past trauma may be triggered by participation in the research and the need for provision of support to participants if this occurred; (ii) the potential for professional boundaries to become blurred if participants became distressed; (iii) the importance of reciprocity and confidentiality for mutual benefit in participation and safety of participants; and (iv) the need to involve both men and women in recognition of gender roles changing in the context of migration and settlement.

There was consensus that participatory approaches are essential in conducting research with refugee communities, and that community engagement needed to be central to the design, content and interpretation of the research. It was also decided to engage community members and other key stakeholders from local services early in the project to assist us in planning community and service engagement strategies, and to advise us on scoping the research to optimise the translational potential of outcomes. The ‘concept’ we aimed to demonstrate was an approach to cross-cultural research that included community engagement and consultation in determining the best ways to ‘hear the voices’ of the Afghan community and build an understanding of how Afghan women and men experience health care during pregnancy, the time of giving birth, and over the first few months after having a baby in Australia.

**Figure 1:**
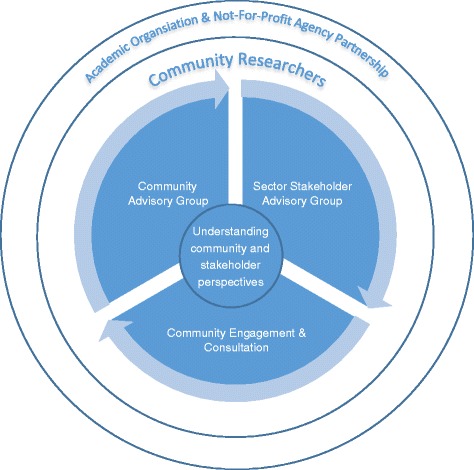
**Participatory framework to involve the Afghan community and local stakeholders.**

The partnership evolved over several months during which arrangements for study governance were discussed and finalised, a set of agreed values and principles to underpin the collaboration, fundamental aspects of the research protocol, resources required to undertake the project, and a formal agreement documenting how we planned to work together. The time taken at this stage of the project to clarify expectations and have ‘big picture’ discussions about ethical issues we were likely to face in implementing the project proved to be important to the ultimate success of the project. A community engagement framework was devised and comprised the following components: appointment of two community researchers to facilitate consultation and engagement of Afghan women and men in the project; establishing a Community Advisory Group to guide the community researchers and other members of the team in carrying out the project; and building into the project an initial phase of community consultation to gauge community attitudes to the idea of the study and inform the study protocol. The framework is summarised in Figure 1. The partnership (all authors) met at key stages of the project, and a smaller project implementation group (JY, ER, FF, SW, SC, PD-P, DC) met regularly to pool ideas and wisdom about how to tackle problems as they arose, and progress the work of the project.

## A framework for community engagement

### The setting and population

Of the total number of people accepted into Australia annually as part of the Humanitarian program, around a third settle in the state of Victoria. Over the past five years people who were born in Afghanistan formed the largest group of migrants to Victoria, with 6,960 settling under the Humanitarian program and a further 1,400 arriving as part of the family-reunion stream [13]. The decision to base the project in Melbourne’s south east region and seek to engage the Afghan community living in this area of Melbourne was based on a number of factors. The region is a major area of settlement of refugees, largely from Afghanistan, Sri Lanka, Iran and Pakistan. Previous research had identified that Afghan women living in this region feel physically and emotionally isolated from care during pregnancy, and men are having to assume a new role caring for their wives at the time of having a baby [14]. Whilst research with fathers is gradually increasing, research with fathers of refugee background remains scarce. This ‘proof of concept’ project provided an ideal opportunity to test engagement strategies and include the perspective of Afghan fathers. Foundation House was working with local residents of Afghan background to identify and provide advice to address community issues. The Afghan advisors had identified ‘maternity’ as an area requiring attention. This local initiative provided an opportunity to work with the Afghan community to explore an issue important to them.

### Employing community researchers

Foundation House’s strong relationship with the Afghan community had evolved over several years of working with community advisors and families. The organisation’s knowledge of the community alerted the research team to a number of issues that needed to be considered and integrated into the project. For example, in the Afghan community it was essential to respect cultural protocols regarding how decisions might be made to take part in research, specifically, the need to inform both men and women about the research, and involve both in making decisions about women’s participation. Employing both female and male community researchers was a strategy adopted to optimise community engagement by facilitating men’s and women’s involvement. Likewise, the strategy was thought to ensure that the voices of the Afghan community were heard authentically by minimising the risk of a researcher of non-Afghan background being viewed as an expert person of authority. In considering this, during the interview process all applicants were presented with case scenarios to assess their sensitivity, articulate their boundaries and the role they would play as a community researcher, rather than drawing on their authority, expertise and status as perhaps viewed by the community.

Often community participation in research projects involves community members volunteering their unpaid time, whilst fulfilling multiple roles such as locating and recruiting study participants and acting as interpreters [15,16]. Although this approach can provide opportunities to individuals from the community that might not otherwise be available [17], the approach adopted for our study was to employ the community researchers as employees of the research organisation, MCRI. A traditional employee recruitment process was conducted and the positions were also advertised through Foundation House networks, community-based agencies and word of mouth via Afghan community networks. The partnership team developed a position description for the role of what was described originally as bicultural research interviewers, including criteria considered essential for the project. These included written and spoken English language fluency and fluency in a key Afghan language (Dari), a demonstrated ability to work with Afghan families and community, and excellent listening and oral communication skills. These skills were considered more important than prior research experience or formal qualifications. Beyond this, the capacity to work across the community irrespective of affiliations (e.g. religious, ethnic, political). Other characteristics included: an inclusive and open-minded attitude; good cross-cultural understanding and being comfortable working in their own Afghan culture and the Australian culture; and the ability to be reflective was also necessary [18]. Short-listing and interviewing of applicants was undertaken by members of the partnership group.

Two community researchers, a female (FF) and a male (SW), were employed. The term ‘community researcher’ was adopted to describe their involvement in all aspects of the project and they were known as this throughout the project. Their roles included assisting in the establishment of a Community Advisory Group (CAG), designing and leading the community consultation, recruiting male and female participants for the research, conducting data collection, assisting with analysis and interpretation of the findings and sharing the findings with the participants and broader community. Both community researchers were born in Afghanistan, had strong connections to their communities and between them spoke four Afghan languages as well as English. Both had degree level qualifications and one had extensive public health experience. This was their first experience of conducting research with the Afghan community in south east Melbourne.

The community researchers worked closely with two experienced researchers (JY, ER) to develop their skills in all aspects of research. They also shared their community knowledge with other members of the research team and deepened their understanding of cultural and ethical issues that needed to be incorporated in the design and conduct of the research. The time frame for project development and implementation was designed to allow time for capacity exchange, with continuous opportunities for group reflection, particularly during data collection. To facilitate and complete the research tasks, the ‘research team’ (JY, ER, FF, SW) met regularly at the office where the community researchers were based, and worked closely on all day-to-day aspects of the project. Office space for the project was provided by Foundation House at their Dandenong office in order for the community researchers to be located in the setting where there the research was being undertaken. MCRI based team members made regular weekly visits to the Foundation House office to work with the two community researchers. Via these processes we were able to identify when the research processes were working well, and when things needed to be done differently. For example, when the team reflected on the interview transcripts early in data collection, it was recognised that some of the more structured interview questions needed to be followed up with more open-ended inquiry to enrich the quality of the data.

Although the intent of participatory research is to include community input in all phases of a research initiative, engagement in the process of data analysis and interpretation of findings, occurs less frequently than in other research phases [19]. The involvement of the community researchers in data analysis was critical. Concurrent coding and discussion of the findings amongst the project team provided several advantages. For instance, the community researchers could see what happened with the ‘data’ that was being collected from participants and how this was used to answer the research questions. This allowed the community researchers to identify where in interviews they could ‘probe’ and inquire more to extract further detail from participants. Further, the participation of the community researchers brought a cultural lens to the interpretation of the results, and generated a depth of understanding that otherwise would not have been possible. For example, the results showed that there was very low knowledge and uptake of local services by all participants. By way of explanation, the community researchers suggested that a person’s non-disclosure was related to feelings of shame. If you disclosed knowing of a service such as counselling it usually meant you were using them and it is well known that mental health issues are stigmatised in the community.

### Establishing project advisory groups

There is increasing recognition that community and stakeholder engagement and participation in the design, planning, implementation, delivery and evaluation of health services will result in service provision more in tune with community needs [20]. To facilitate the engagement of the Afghan community and local services, two advisory groups were established early in the project.

A Community Advisory Group (CAG) was established to facilitate community engagement. The CAG included Afghan women and men from the local community from three Afghan ethnic groups - Hazara, Pashtun and Tajik. Community advisors were identified and invited to participate by the community researchers and via existing networks from previous Foundation House projects. The roles of the Community Advisory Group were to: (i) provide community perspectives to ensure the research questions and processes for data collection were right; (ii) contribute to interpretation and dissemination of the findings; (iii) facilitate further consultation and community engagement; and (iv) provide a conduit between the partnership team and the community. CAG members were provided with supermarket gift vouchers as a token of thanks for their role and participation. The CAG met three times over the course of the project.

A Sector Stakeholder Advisory Group (SSAG) was also established and comprised key stakeholders from relevant policy sectors (health, early childhood, local government) and local health services. The role of this group was to (i) provide advice about key questions from a service and policy perspective, and (ii) ensure that the research findings were positioned to influence policy, organisational change and practice. The SSAG met three times during the course of the project; twice during the implementation phase, and once after the final report of the project was completed. Joint agreement on the research aims from the start of the project ensured that the scope of what could be achieved within the given time frame and budget was feasible, and facilitated a sense of joint ownership. The engagement of diverse stakeholders as advisors to the project also proved to be a successful knowledge exchange strategy. In the final stakeholder advisory group meeting there was a collective response to the study findings with desire to address the inadequacies highlighted by the research. Others have also reported these as important considerations for establishing diverse partnerships of this kind embarking on joint-projects [21].

Also informed by SSAG members, a complementary study invited local stakeholders including health care professionals, managers and community workers to participate in interviews and focus groups in order to inform the proof of concept study. Thirty-four people participated, including midwives, general practitioners, maternal and child health nurses, obstetricians and other community and bicultural workers.

### Conducting community consultation

The primary goal of the consultation was to gauge community interest in the research, and seek views about ways to engage families and hear the voices of Afghan women and men about their experiences of Australian maternity and early childhood services to inform the design of the research. Specifically, the consultation aimed to: (i) establish a profile of the project in the Afghan community utilising existing channels/resources and expanding new community contacts and networks; (ii) seek community feedback in relation to maternity and postnatal health care to assess the importance and relevance to the Afghan community and any other important issues that should be considered in the study; (iii) explore approaches that would be suitable for collecting data from both women and men and that are culturally appropriate and mindful of potentially sensitive issues; and (iv) identify settings or access points for approaching eligible participants for the research phase.

In meeting these aims, the importance of building relationships between community members and the community researchers was essential. A snow ball sampling strategy was used to identify and approach key people through existing professional and personal networks of the research team, partnership members, and members of the CAG to assist in the organisation of consultation sessions. The community researchers used a variety of approaches via telephone, email and on-site visits to make contact with community members, develop relationships with key gatekeepers, organise consultation sessions and encourage people attending the consultation meetings to participate in the research project.

Ninety-four Afghan women and men participated in 12 groups (7 women’s groups and 5 men’s groups, there were no mixed gender groups) and 3 telephone consultations. Most of the women’s groups were typically pre-existing groups such as playgroups, whilst men were approached and invited to participate in once-off groups. Of the Afghan ethnicities, Hazara, Tajik and Pashtun women and men were represented and most had experienced having a baby in Australia. Some consultation participants were grandparents who reflected on their child/children’s experiences of care during and after pregnancy. Further demographic details were not collected at this stage as the community researchers were not certain what information would be appropriate and acceptable to ask in group settings. The consultation was fruitful for the community researchers as they were able to identify a pool of eligible participants for the research phase. Most of the community consultation sessions were lively with much animated discussion about the research and about the issues of importance to the community. Whilst those consulted stressed the importance of confidentiality in asking women and men about issues related to having a baby, many freely talked about what may be considered sensitive topics, for example mental health issues. It was clear that in asking Afghan women and men about potentially sensitive issues related to having a baby, individual interviews were preferable to focus groups. The importance of ensuring confidentiality was raised by many during consultations adding further support for interviews.

The consultation identified that the time of having a baby can be a stressful and challenging time for Afghan families. In particular, consultation with Afghan women and men revealed that: access to information during pregnancy and early childhood was problematic; the gender of care providers and interpreters during pregnancy appointments was of a major concern; and access to interpreters in their own dialects was also very important. Other issues highlighted by Afghan women included: relationship problems, access to care, cross cultural differences, maternal depression, family violence and experiencing personal discrimination by service providers. Men discussed: unemployment; accessing Centrelink benefits (social security); appropriate housing; family reunion visas; understanding the health system and challenges associated with supporting their partners to use services. Participants in existing women’s groups spoke freely. Knowing each other, shared language, not asking personal questions or audio recording the discussions may have been factors in people’s comfort in participating. Groups where women were not known to each other were much more reserved.

The consultation proved valuable as a community engagement strategy and in ensuring that community members felt that the people conducting the study took appropriate steps to understand the issues and concerns they identified. A key finding from the consultation was that people in the Afghan community were comfortable participating in focus groups to discuss general issues, despite the preference for interviews to discuss sensitive issues. Based on this experience there appear to be benefits in combining both quantitative (i.e. interview style semi-structured questionnaire) and qualitative (e.g. focus groups/in depth interviews) methods in the conduct of research with Afghan and perhaps other refugee background communities. Such an approach is likely to be acceptable to both women and men and to allow in-depth exploration of issues.

## The research – interviews with Afghan women and men

As outlined, the community engagement framework was implemented with careful consideration of ethical issues relevant to undertaking research with people of refugee background. To this end, a protocol was developed with input from all partner members, to guide the community researchers in responding to participants who may become upset or distressed, or in the event that they were concerned about the welfare of the participant or a family member. The joint-process of developing the protocol allowed for clarification of the community researcher’s role and the boundaries associated with their role whilst acknowledging the situations in which support should take place and how they could provide this. The protocol also outlined the role of the community researchers in responding to requests from participants for information, advice and support and included a reference to a directory of local services. The directory was used on several occasions to provide participants with information about accessing the local services.

Ethical approval was granted by both the Victorian Foundation for the Survivors of Torture and the Royal Children’s Hospital Human Research Ethics Committees.

### Recruitment and sample

Recruitment was undertaken by the community researchers (FF, SW). They worked with the CAG and other community members who assisted with the consultation, contacted local services and community groups to identify sources of potential participants and invited women and men to participate. A postcard with information about the study and information about how to take part and the contact details of the community researchers, in Dari and English, was distributed. The information was also printed in the local Afghan community newspaper over a four week period.

During the consultation the community researchers occasionally made arrangements for an interview with those interested in participating. This was acceptable for men. Women were more likely to want to seek the permission of their husbands before committing to participation. In several instances multiple contacts were required so that women felt adequately informed and comfortable about participating.

At the time of the interview women and men were asked to consent to the research, either in writing, or verbally. Recognition that in refugee populations there can be a fear of ‘authority’ and/or limited literacy in one’s own language and signing forms can be confronting, to alleviate this the option of verbal consent was provided, whereby the participant would repeat a short script that was digitally recorded then transferred to a CD for storage. Interviews were undertaken by the community researchers in the language preferred by the participant, usually Dari. Participants were also asked their preference for where the interview should take place (e.g. home, community meeting space, Foundation House) and if arrangements needed to be made for transport and/or childcare. Participants were given a $50 supermarket/department store gift voucher to thank them for taking part.

Sixteen women and 14 men participated in individual interviews. All were born in Afghanistan, had a baby that was around 4–12 months. Participants were diverse and reported their background as Hazara, Tajik, Pashtu, Afghan or Sadath, spoke several Afghan languages and had lived in Australia anywhere from a few months to more than six years.

### Data collection and analysis

Design of the interview topic guide commenced with the community consultation and CAG and SSAG members to identify key outcomes of interest. The interview guide was piloted with four Afghan women and two Afghan men. Minor amendments were made primarily to reduce the number of questions. All interviews used the same topic guide. All interviews were audio-recorded and the community researchers documented responses and took detailed field notes at the completion of the interviews. The community researchers transcribed all interviews to English (80% of interviews were conducted in Dari or other Afghan language). Once transcribed, the community researchers cross-checked each other’s transcription documents by listening to the audio recordings and discussing any amendments. Thematic analysis was applied whereby transcripts were coded, codes were then organised into logical categories and overall themes became evident [22].

### Confirmation and dissemination of the findings

Participants in the community consultation told us that hearing about the research findings was important to them and the broader community. They indicated that sharing information was best done verbally, and there were also some suggestions for printed summary reports in Dari. Suggestions for the best avenues to hear about the findings included: community events, returning to the consultation venues, from health care providers such as maternal and child health nurses, the Afghan Association (local ethno-specific organisation) and ethnic media – radio and newspapers. The community researchers recommended that a community forum at Foundation House, incorporating a shared lunch, would be a suitable avenue for sharing the findings. All participants and key community members who helped with the consultation were invited, in all about 30 people. The community researchers provided an overview of the findings in Dari and allowed opportunities for discussion. Community members who attended the forum: enquired about ‘what changes in health services will happen now as a result of this research?’; confirmed that Afghans were unaware of how social issues impact on health; and complimented the research team on ‘a thorough research project that truly heard the voices of local Afghan people’. The research team continue to work with the community advisors to share the results with the broader community through avenues including playgroups and women’s groups.

For health services, policy makers and other stakeholders a final project report was produced documenting the methods, key findings and implications for health policy and services. A well-attended launch of the report was held at Foundation House. All CAG and SSAG members were invited to attend and each CAG member was provided with a small gift of thanks. The research team have also had opportunities to present the findings at academic conferences as well as in meetings with service providers and managers.

## Learnings from the proof of concept study

The partnership between the two agencies shaped the way in which all aspects of the research project were designed and implemented. This co-production of research evidence was instrumental in ensuring meaningful and useful outcomes from the research to inform strengthening of services for families of refugee background. In addition, the fruitful partnership fostered trust and built capacity, providing a strong foundation for future work. As others have identified, appropriate funding is required to support reciprocal partnership approaches [23]. Significant resources were committed by the partners, in particular, the commitment of time to attend regular meetings, active involvement in development of study protocols, interpretation of study findings, and investment in capacity exchange as a core activity of the project.

This is the first research of its kind, nationally and internationally, to conduct a community-based maternal and child health research study with an Afghan refugee background population. Most mainstream research does not make provision for the inclusion of culturally and linguistically diverse (CALD) migrant communities. There is recognition that these often ‘hard to reach’ communities are excluded because conventional research methods, such as written surveys, are simply not appropriate and the subsequent study limitations are attributed to lack of financial resources [24]. Furthermore, people of migrant and refugee backgrounds may be reluctant to participate in research because it is unfamiliar to them, coupled with apprehension about providing personal information to researchers who may be perceived to be associated with governmental agencies [25]. As noted in one of the community researchers field notes:

*The Afghan community is less familiar with the term “research” and it is often taken as “investigation” therefore, to familiarise members of the community with the research project, informal visits were made prior to the formal consultation sessions to meet the women and introduce the project. Participants were highly appreciative of the consultation process and having an opportunity to contribute to the direction of the research. Most of the participants have never been part of a research project or have never heard about the research process.*

The employment of ‘community researchers’ to consult with migrant communities about the design and conduct of research, and involvement of community researchers in all aspects of the design, conduct and interpretation of research differentiates our study from many other studies that have sought to engage with migrant communities [26]. Working with the community researchers facilitated a process of jointly negotiating cultural subtleties and ethical strategies with the community at every phase of the research process [27]. The benefits included a robust community consultation to elicit the issues that were important to the community to explore in the research, the most appropriate methods, and considerations for what the research team needed to be mindful of in conducting this project. Rarely are the voices of fathers included in maternal and child research, this proof of concept project provided an opportunity to explore their interest, engagement and participation. Employing both male and female community researchers was a key strength of this study. In addition, the community researchers’ active role in the data analysis enhanced the reliability and validity of the findings by providing their cultural knowledge and insight [28]. Over the duration of the research project the community researchers’ skills developed tremendously. They flourished in both the community-based and academic environments. This was evident in their confidence to explain research processes to participants such as informed consent, their ability to probe and use prompting questions in interviews and contribution to the coding and categorising of transcript data. The involvement of the community researchers in all key elements of the research process helped to bridge the unequal relationships – between researchers and research participants, and between community members invited to participate in research - which may otherwise not be recognised in research processes. Project collaborators need to be prepared to source specific funding to appoint suitably matched staff on cross-cultural research such as this, in terms of their multi-language ability and knowledge of their community [29]. The specific roles played by the community researchers were fundamental to the success of the project, and production of meaningful outcomes from the research. Importantly, the community researchers were not just intermediaries between the researchers and the community whose participation was sought. Their multiple and ongoing contact with the Afghan community living in the south east of Melbourne, facilitated an understanding of research and what can be achieved within the scope of a research project. Reflecting on the community consultation, one of the community researchers noted the need to navigate community expectations:

*There was enthusiasm for the research and hope for minor changes in health care system that could fulfil their needs in the best possible way; however, I needed to clarify that whilst the research hoped to inform change this was not guaranteed as a direct outcome of this project.*

Much is documented about the usefulness of community-based participatory methods for involving typically ‘hard to reach’ people to recognise and include their often excluded voices in research [15,30]. Done properly, participatory methods can benefit community members, health care professionals and managers, policy makers and researchers alike. Participatory methods can create bridges between researchers, stakeholders and communities, through the sharing of knowledge and experience. Such inclusive research practices, although innovative are rarely documented in detail for others to learn *how* to conduct research of this kind. This project was designed to ascertain the feasibility and value of participatory methods to engage women and men of refugee background, in this case from the Afghan community. Planning the community engagement framework required consideration of the community’s geographic, demographic and cultural characteristics, as well as the community’s interest and desire to take part. The usefulness of the approach included building on the existing local community networks, as well as making the most of opportunities for capacity building with local agencies and community members by strengthening relationships, leadership skills and knowledge of all those involved in the Community Advisory Group, and the community and health professional/stakeholder participants and their agencies. Migrant Afghan women have previously participated in research projects [30,31], but there are few examples of men participating and have their voices documented. The fact that numerous men participated in the consultation and the research interviews is testament to the value of the methodology employed.

Overall, this process has demonstrated the value of partnerships and community engagement. These findings about method and process are generalizable to other refugee, culturally diverse and vulnerable populations and can be applied by others whom are implementing cross cultural research.

Although this was a time-limited research project, the commitment to continuous knowledge exchange between the partners, and development of relationships with key policy and service stakeholders led to the development of a major new project titled *Bridging the Gap: Partnerships for change in refugee child and family health* [32]. This project involving MCRI, Foundation House and nine other partner organisations in implementing and evaluating a program of quality improvement initiatives to improve the accessibility and responsiveness of maternity and early childhood health settings in Melbourne’s south eastern and western suburbs – both regions have significant refugee background populations.

In summary, inclusive research strategies that address power imbalances in research, and diversity within communities, are necessary to obtain the evidence required to address health inequalities in vulnerable populations. Such an approach involves mindfully adapting conventional research processes to ensure that studies have regard for the advice of community members about the issues that affect them. Researchers have much to gain by committing time and resources to engaging communities in a reciprocal way in research processes.

## References

[1] Fiscella K, Shin P (2005). The Inverse Care Law: Implications for Healthcare of Vulnerable Populations. J Ambul Care Manage.

[2] Cooper LA, Beach MC, Johnson RL, Inui TS. Delving below the surface. Understanding how race and ethnicity influence relationships in health care. J Gen Internal Med. 2006;21 Suppl 1:S21-7. 10.1111/j.1525-1497.2006.00305.x PMC1484840 16405705

[3] Herceg A. Improving health in Aboriginal and Torres Strait Islander mothers, babies and young children: a literature review. In Book Improving health in Aboriginal and Torres Strait Islander mothers, babies and young children: a literature review (Editor ed.^eds.). City: Commonwealth of Australia; 2005

[4] Hunt J (2006). Trying to make a difference: a critical analysis of health care during pregnancy for Aboriginal and Torres Strait Islander women. Australian Aboriginal Studies.

[5] Victorian Refugee Health Network: Maternity care for women from refugee backgrounds. Discussion paper. In: Book Maternity care for women from refugee backgrounds. Discussion paper. (Editor ed.^eds.). City: Victorian Foundation for Survivors of Torture; 2012.

[6] Kennedy P, Murphy-Lawless J (2003). The maternity care needs of refugee and asylum seeking women in Ireland. Feminist Rev.

[7] Bulman KH, McCourt C (2002). Somali refugee women's experiences of maternity care in west London: a case study. Crit Public Health.

[8] Small R, Lumley J, Yelland J (2003). Cross-cultural experiences of maternal depression: associations and contributing factors for Vietnamese, Turkish and Filipino immigrant women in Victoria, Australia. Ethn Health.

[9] Shafiei T, Small R, McLachlan H (2012). Women’s views and experiences of maternity care: a study of immigrant Afghan women in Melbourne, Australia. Midwifery.

[10] Cheng I-H, Russell G, Bailes M, Block A. An evaluation of the primary healthcare needs of refugees in South East Metropolitan Melbourne. In: Book An evaluation of the primary healthcare needs of refugees in South East Metropolitan Melbourne (Editor ed.^eds.). City: Southern Academic Primary Care Research Unit; 2011.

[11] Riggs E, Davis E, Gibbs L, Block K, Szwarc J, Casey S (2012). Accessing maternal and child health services in Melbourne, Australia: Reflections from refugee families and service providers. BMC Health Serv Res.

[12] Yelland J, Riggs E, Wahidi S, Fouladi F, Casey S, Szwarc J (2014). How do Australian maternity and early childhood health services identify and respond to the settlement experience and social context of refugee background families?. BMC Pregnancy Childbirth.

[13] Settlement Reporting Facility [http://www.immi.gov.au/settlement/srf/]

[14] Rintoul A. Understanding the mental health and wellbeing of Afghan women in South East Melbourne. In: Book Understanding the mental health and wellbeing of Afghan women in South East Melbourne (Editor ed.^eds.). City: Monash University and Foundation House; 2009.

[15] Israel B, Schulz A, Parker E, Becker A (1998). Review of Community-Based Research: Assessing Partnership Approaches to Improve Public Health. Annu Rev Public Health.

[16] Centre for Culture Ethnicity and Health. Bilingual Staff Research Project Report. In: Book Bilingual Staff Research Project Report (Editor ed.^eds.). City; 2008.

[17] Flicker S, Travers R, Guta A, McDonald S, Meagher A (2007). Ethical Dilemmas in Community-Based Participatory Research: Recommendations for Institutional Review Boards. J Urban Health.

[18] Small R, Yelland J, Lumley J, Rice P (1999). Cross-cultural research: trying to do it better. 1. Issues in study design. Aust N Z J Public Health.

[19] Cashman SB, Adeky S, Allen AJ, Corburn J, Israel BA, Montano J (2008). The Power and the Promise: Working With Communities to Analyze Data, Interpret Findings, and Get to Outcomes. Am J Public Health.

[20] Buckskin M, Ah Kit J, Glover K, Mitchell A, Miller R, Weetra D (2013). Aboriginal Families Study: a population-based study keeping community and policy goals in mind right from the start. Int J Equity Health.

[21] Hinchcliff R, Greenfield D, Braithwaite J (2014). Is it worth engaging in multi-stakeholder health services research collaborations? Reflections on key benefits, challenges and enabling mechanisms. International J Qual Health Care.

[22] Green J, Willis K, Hughes E, Small R, Welch N, Gibbs L (2007). Generating best evidence from qualitative research: the role of data analysis. Aust N Z J Public Health.

[23] Mitchell P, Pirkis J, Hall J, Haas M (2009). Partnerships for knowledge exchange in health services research, policy and practice. J Health Serv Res Policy.

[24] NHMRC. Cultural Competency in Health: A guide for policy, partnerships and participation. In: Book Cultural Competency in Health: A guide for policy, partnerships and participation (Editor ed.^eds.). City: Commonwealth of Australia; 2005.

[25] Gibbs L, Abebe M, Riggs E, Waters E, Seidell J, Swinburn B (2009). Working with minority groups in developed countries. Community-based obesity prevention: evidence, practice and policy.

[26] Riggs E, Gussy M, Gibbs L, van Gemert C, Waters E, Priest N, et al. Assessing the cultural competence of oral health research conducted with migrant children. Community Dent Oral Epidemiol. 2014;42(1):43-52. 10.1111/cdoe.12058 23869652

[27] El-Jardali F, Lavis J, Moat K, Pantoja T, Ataya N (2014). Capturing lessons learned from evidence-to-policy initiatives through structured reflection. Health Res Policy Syst.

[28] Ellis BH, Kia-Keating M, Yusuf SA, Lincoln A, Nur A (2007). Ethical Research in Refugee Communities and the Use of Community Participatory Methods. Transcult Psychiatry.

[29] Aldridge J (2014). Working with vulnerable groups in social research: dilemmas by default and design. Qual Res.

[30] Lindgren T, Lipson JG (2004). Finding a Way: Afghan Women’s Experience in Community Participation. J Transcult Nurs.

[31] Iqbal N, Joyce A, Russo A, Earnest J (2012). Resettlement experiences of Afghan Hazara female adolescents: a case study from Melbourne. Australia. Int J Popul Res.

[32] Bridging the Gap. Partnerships for change in refugee child and family health [http://www.mcri.edu.au/research/research-projects/bridging-the-gap/]

